# Impact of Automatic Query Generation and Quality Recognition Using Deep Learning to Curate Evidence From Biomedical Literature: Empirical Study

**DOI:** 10.2196/13430

**Published:** 2019-12-09

**Authors:** Muhammad Afzal, Maqbool Hussain, Khalid Mahmood Malik, Sungyoung Lee

**Affiliations:** 1 Department of Software Sejong University Seoul Republic of Korea; 2 Department of Computer Science and Engineering Oakland University Rochester, MI United States; 3 Department of Computer Science and Engineering Kyung Hee University Yongin Republic of Korea

**Keywords:** data curation, evidence-based medicine, clinical decision support systems, precision medicine, biomedical research, machine learning, deep learning

## Abstract

**Background:**

The quality of health care is continuously improving and is expected to improve further because of the advancement of machine learning and knowledge-based techniques along with innovation and availability of wearable sensors. With these advancements, health care professionals are now becoming more interested and involved in seeking scientific research evidence from external sources for decision making relevant to medical diagnosis, treatments, and prognosis. Not much work has been done to develop methods for unobtrusive and seamless curation of data from the biomedical literature.

**Objective:**

This study aimed to design a framework that can enable bringing quality publications intelligently to the users’ desk to assist medical practitioners in answering clinical questions and fulfilling their informational needs.

**Methods:**

The proposed framework consists of methods for efficient biomedical literature curation, including the automatic construction of a well-built question, the recognition of evidence quality by proposing extended quality recognition model (E-QRM), and the ranking and summarization of the extracted evidence.

**Results:**

Unlike previous works, the proposed framework systematically integrates the echelons of biomedical literature curation by including methods for searching queries, content quality assessments, and ranking and summarization. Using an ensemble approach, our high-impact classifier E-QRM obtained significantly improved accuracy than the existing quality recognition model (1723/1894, 90.97% vs 1462/1894, 77.21%).

**Conclusions:**

Our proposed methods and evaluation demonstrate the validity and rigorousness of the results, which can be used in different applications, including evidence-based medicine, precision medicine, and medical education.

## Introduction

### Objective and Contributions

Personalized health care and wellness management have rapidly grown during recent years because of the increase in data influx, the development of innovative tools, and the advancement of artificial intelligence techniques. These innovations can engage patients and offer additional modalities in the treatment of chronic diseases [1]. In addition, with the advent of the next-generation sequencing and the widespread use of electronic health records (EHRs), clinicians and researchers have the opportunity to have a wealth of data and the precise characterization of individual patient genotypes and phenotypes [2]. It is now evident that the research on internet health information–seeking behavior is on the rise [3].

Furthermore, people’s interest in seeking the support of scientific research evidence is increasing daily for their level of satisfaction over medical decisions or advice, and it keeps them aware of the research about the matter. Clinicians seek for external evidences to make informed clinical decisions, particularly when internal evidences (information derived from unicenter data) are insufficient because of lack of required data. Likewise, medical researchers and students are interested in the external evidences to educate themselves on the substance of a medical problem, whereas the patients could use such evidences for their own awareness and comparative analysis of available treatments. Fortunately, an overwhelming amount of biomedical information is available in the form of scientific publications, which can be retrieved to support the process of medical decision making and for self-awareness. PubMed, which is a search engine for biomedical literature, can provide access to a set of more than 27 million articles from more than 7000 journals, including full text for about 4 million of these articles [4]. However, the current process of retrieving research publications from the external biomedical literature is a daunting task and is largely done manually, which requires not only a high level of expertise but also time and money. As the demand for evidence-based medicine (EBM) is increasing, it is important to lower the costs to identify and evaluate the best evidence. Little has been done to improve the overall efficiency of curating the quality evidences automatically from the biomedical literature until recently. One of the major challenges in this regard is to design the search query from the input information and to embed the user context in an automatic and intelligent manner to save time and cost. In addition, the low quality of the articles from where the evidence is retrieved for the decisions adds further to the challenge of an automated acquisition of evidence. Moreover, the results are summarized and ranked majorly with manual efforts.

In this paper, we contributed to the design of a comprehensive framework architecture to achieve the goal of curating biomedical literature and mining data from scientific publications to construct precise evidence to assist medical practitioners, researchers, medical students, and patients in the clinical decision-making process. The proposed framework consists of several methods for automating the process of biomedical curation. The main contributions of this paper are as follows:

- It presents the design of a comprehensive framework for biomedical literature curation. It describes proposed architecture in detail, which includes designs for methods of well-built automatic query construction, evidence quality recognition, and article summarization and ranking.
- It describes the proposed process of the construction of a well-built query. We designed a set of methods and guidelines to construct a well-structured question from the input information in a standard format for a better user understandability and content categorization.
- It presents the design of proposed extended quality recognition model (E-QRM) that identifies scientifically sound publications on the basis of content rigorousness. We developed and compared a set of machine and deep learning (DL) models with a higher level of precision as evaluation criteria.
- It offers methods for contextual ranking and summarization. We designed a cross-context interpretation model for ranking the publications based on the context captured from the input information, the user of the system, and the articles that are retrieved. In addition, we propose a conceptual model for summarization of the results based on input information.

### Background and Motivation

For evidentiary support, medical professionals mostly rely on the publicly available searching services, such as PubMed [5], Google, UptoDate [6], and other search engines. These search engines are reliable, but they need to be integrated with a health care information system (HIS) in a way to make the process of evidence retrieval seamless and meaningful. In addition, an HIS is required to evaluate the retrieved evidence for quality rather than relying on a search engine’s built-in evaluation mechanisms.

Some of today’s HISs are equipped with the knowledge base (KB) of a clinical decision support system (CDSS), which provides additional support to automate the evidence retrieval from external sources in following ways: (1) it aids to automate query construction process for evidence retrieval by offering knowledge rules that consist of patient information with established logical connections and (2) it assists to enrich query for evidence with metadata such as the purpose or the query type information to improve the quality of evidence extraction. The query type information shows the purpose for which a CDSS is developed, such as a treatment plan or diagnosis recommendations.

**Figure 1:**
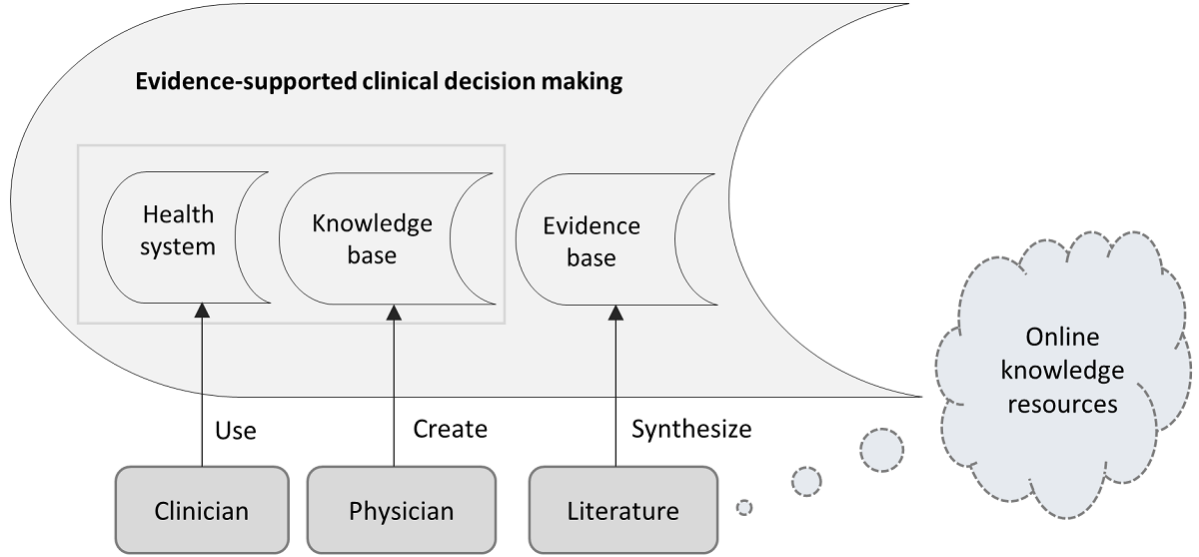
Clinical decision support system knowledge base linkage with evidence base synthesized from biomedical literature.

Figure 1 shows the interaction among a health system, KB of CDSS, and the system for extraction of external evidence resources. The health system manages the patient records to be used by the clinician, and the KB of a CDSS is created with the support of expert clinicians either through directly authored rules or the machine learning (ML)–based data-driven approaches [7]. The evidence-based subsystem shows the appraised evidence synthesized from the literature through the automatic methods of acquisition and appraisal. In this study, we proposed a comprehensive framework to combine the abovementioned processes, particularly, the evidence acquisition and evidence appraisal to facilitate the clinical decision making. The proposed methodology uses the information contents from a health system and the KB of CDSS for the query construction to search and retrieve relevant research papers from the literature to support the evidence-based practice (EBP). The EBP and the CDSS have long been used in the clinical domain to enhance clinical efficacy. The EBP and the CDSS share clinical expertise as a source of data. The EBP uses the clinical expertise along with the research evidence and other factors for a clinical decision. A CDSS KB is the representation of clinical expertise of 1 or more clinical experts. The EBP is defined as “the conscientious, explicit, and judicious use of current best evidence in making decisions about the care of individual patients” integrated with clinical expertise and patient values to optimize outcomes and quality of life [8].

### Role of User Context in Query Construction

For any evidence-based system to work efficiently, the context of the domain plays a crucial role. The context provides the features for query generation to seek the relevant information from external sources. The source and format of the data are crucial to consider automatic or semiautomatic query generation. Cimino [9] presented the idea of Infobuttons and Infobutton Manager, which attempt to determine the information needs based on the user context. Infobuttons are mainly topic-specific questions with a facility for the users to tune the query more toward the context. CDAPubMed [10] is a browser extension that aims to provide a tool to semiautomatically build complex queries. It provides additional information to the contents of the EHR to improve the biomedical literature searches. A platform called ProvCaRe [11] has the provision for search and query operations on provenance metadata to enable reproducibility of research articles. There are other approaches described in the studies by Bakal et al [12] and Sahoo et al [13] that use semantic patterns over biomedical knowledge graphs for treatment and causality predictions and semantic provenance to apprehend high-quality domain-specific information using expressive domain ontologies.

### Related Work on Finding High-Quality Articles in the Literature

A decent set of approaches is available that had improvised the results of literature searching with respect to quality of studies. The PubMed Clinical Queries (CQ) [14] is one of the most prominent endeavors to retrieve scientifically sound studies from the biomedical literature. Afterward, supervised ML approaches were introduced mainly to improve the precision of the results in terms of quality checking for methodological rigorousness. Similarly, to find high-quality papers in MEDLINE, Wilczynski et al [15] developed CQ filters, which were later adapted by PubMed for use as CQ. The data collection used in the CQ filters is annotated across the following 4 dimensions: the format, the human health care, the purpose, and the scientific rigor. The experimental studies [16,17] introduced ML (supervised learning) classification models to differentiate between the methodologically rigorous and the nonrigorous articles. In an article about evidence quality prediction [18], the authors addressed the problem of automatic grading of evidence on a chosen discrete scale. The authors experimented many features, such as publication year, avenue, and type to evaluate the quality of the evidence. They found that the publication type is the most eminent feature to consider for evaluation of the evidence quality results. A DL neural network known as the Convolutional Neural Network approach [19] was very recently tried to further improve accuracy over the existing approaches of PubMed CQ and McMaster’s text word search in terms of precision.

### Limitation of the Existing Approaches

The existing approaches discussed mainly focus on the automation of evidence processing to overcome the central problem of time spent on searching while practicing EBM. The inclusion of the research evidence in clinical decisions varies with respect to domain context and objective. Conceptually, the evidence adaption follows the same 5As cycle as mentioned in the study by Leung [8]; however, implementation makes the scenario different. A user in a clinical setup with a CDSS implementation needs to approach the evidence differently than a user who does not have a CDSS implementation. The dataset selection, the feature engineering, and the context awareness bring uniqueness to the approach and pose challenges at the same time. The objective of this study was to circumvent the issues of efficient searching in the biomedical literature to find evidentiary articles that are qualitative and fit-to-context in the user scenario.

## Methods

### Overview

**Figure 2:**
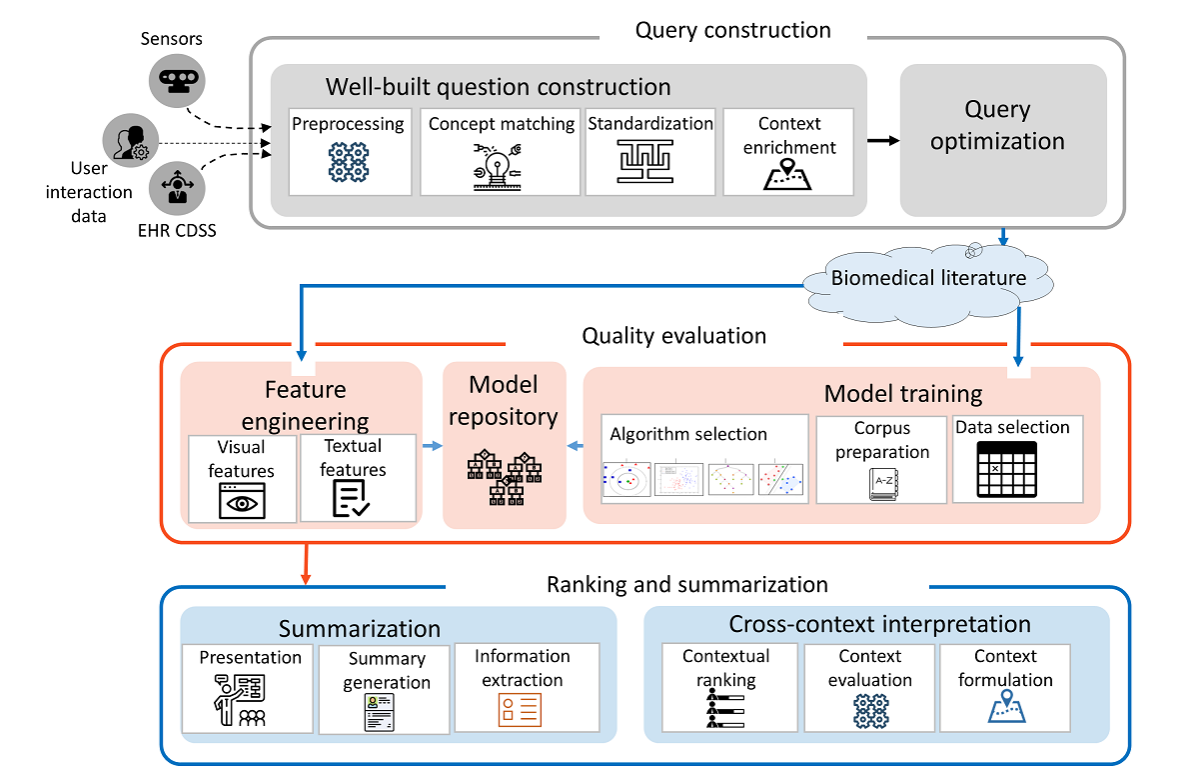
The conceptual diagram of the proposed biomedical literature curation framework. CDSS: clinical decision support system; EHR: electronic health record.

To achieve the research goal of curating and mining data from scientific publications in biomedical literature, we designed a coherent and comprehensive architecture of the framework, which is depicted in Figure 2. The architecture is divided into 3 layers to accommodate the necessary functions of connecting a health system with the scientific research. In the first layer, an optimized query is constructed in a well-built form from the input data streams. In the second layer, the quality is evaluated with data-driven approaches that include a ML or DL algorithm, which is meaningfully selected for the input set of parameters and the data requirements. Finally, in the third layer, the scientific research articles that have been evaluated for quality are summarized and ranked according to the user context to bring an article to the top, such that it is not only relevant and qualitative but also contextually viable and applicable.

### Query Construction

**Figure 3:**
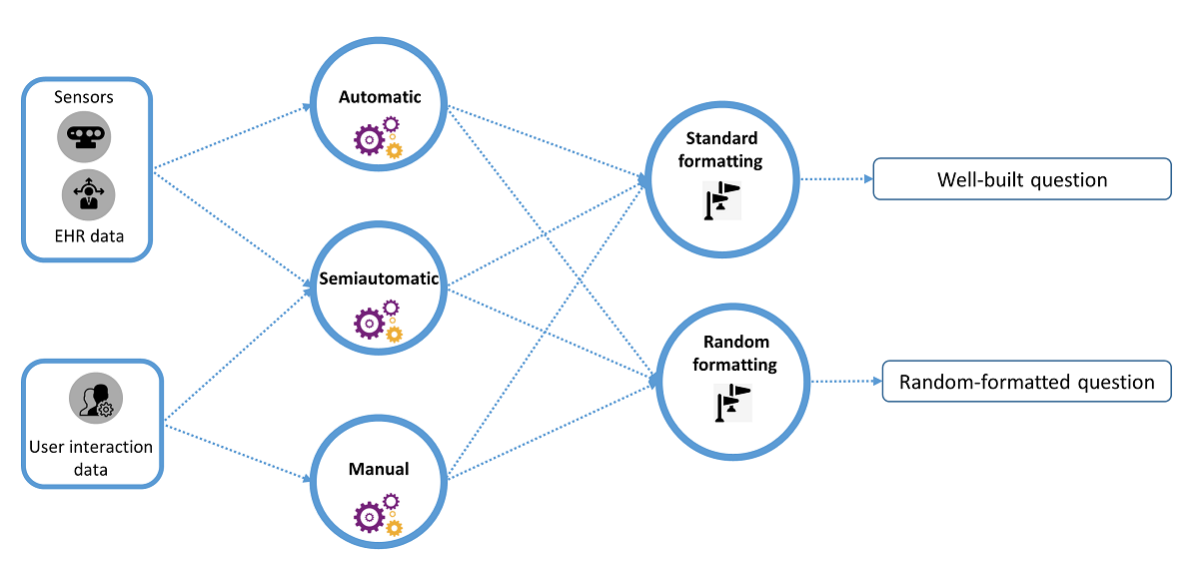
Query construction strategies. EHR: electronic health record.

The query construction is a widely studied and multiaspect topic. One aspect concerns the type of query, which could be manual, semiautomatic, or automatic. Other important aspects include the input data, the context, and the environment of the user. Finally, the query format and the structure could be either just random or well built. Here, we provide a summary of different query construction strategies and recommendations for an efficient strategy from the input clinical information. As shown in Figure 3, there are multiple paths to construct a final query. As examples, we discuss a few popular strategies in Table 1 that were and are in practice or envisioned in this study as a potential futuristic strategy.

**Table 1:** Descriptions of different query construction strategies.

Strategy	Description	Positives	Negatives
The automatic well-built question	In this strategy, a final question is constructed without human intervention. The raw data are acquired meaningfully from the patient information stored in the patient record, and they are associated with the rules of the clinical decision support system or curated from the sensor devices. The acquired elements of the data are transformed autonomously to a well-built or standard format.	An efficient method that does not involve a human to write the query terms; it is easily understandable by the user because of the well-built format; it is comparatively straightforward to summarize the retrieved contents.	Achieving accuracy needs extra effort at the beginning; the design of the intelligent methods is required to correctly place the terms in the appropriate place of a standard or well-built structure.
The automatic random-formatted question	The input part is the same as in the first strategy, and the ingredients of the query are automatically acquired from the input sources. However, they are placed randomly without arranging in a specific format.	It is an efficient method because a human is not required to write the query terms; no effort is required to place the query terms in the required slots.	It is less understandable by the user because of the randomly placed terms; the interpretation and the summarization of the retrieved results will be a daunting task.
The semiautomatic well-built question	The acquisition of the query terms from the input source may be semiautomatic, and human involvement will be necessary to complete the missing section. In addition, placing the terms in the required slots of a standard structure will need human assistance.	Trustworthiness is higher than automatic because of the user’s involvement; an edge in ranking and summarization of the retrieved results.	It is expensive in terms of time because a user will still be required to complete the query contents and the structure.
The semiautomatic random-formatted question	The input acquisition is partially automated. The arrangements of the query terms are random.	The trustworthiness is high.	The interpretation of the query terms and the summarization of results will be a problem.
The manual well-built question	In this strategy, a human is involved thoroughly to write all the contents of a query in a specified structure.	The trustworthiness and a better interpretation of the query terms, the ease in ranking, and the summarization.	It is time consuming; it is hard for naïve users to write complex queries.
The manual random-formatted question	All the contents of the query are written by humans without arranging them in a specific format.	The trustworthiness is high because all the terms are written by the humans.	It is time consuming; it is hard for naïve users; the interpretations, the ranking, and the summarization issues.

In this study, our main focus is the first strategy, which involves constructing an automatic well-built question. We have chosen to formulate the query in Patient/problem, Intervention, Comparison, and Outcome (PICO) format [20] from the input data, which included the patient structured information and the knowledge rules. PICO has a well-structured format, which differentiates different parts of a clinical question in more applicable parts that are easily understandable for the clinicians and other users. It also helps to determine the context of the question. In addition, the structure is helpful to summarize and rank the retrieved articles.

### Input Acquisition and Preprocessing

The contents are preprocessed based on the nature of the input. This section summarizes the guidelines for the developers and the implementers of the representative scenarios. These scenarios represent automatic queries constructed from the following 3 types of inputs: patient record, CDSS rules, and sensory data.

#### Scenario 1: An Automatic Query Construction From a Patient Record of the Electronic Health Record Data

**Figure 4:**
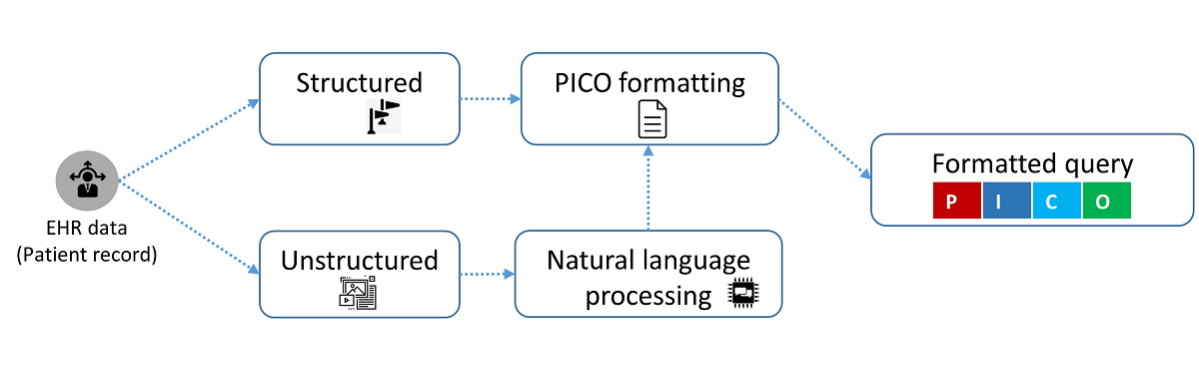
The flow of Patient/problem, Intervention, Comparison, and Outcome query construction from the patient record of the electronic health record data. EHR: electronic health record; PICO: Patient/problem, Intervention, Comparison, and Outcome.

There are 2 possibilities, which involve the data being in a structured or an unstructured format. If the data are structured with assigned labels, they are placed in the target P, I, C, and O sections accordingly. However, if the data are in an unstructured format, then an additional step of the natural language processing (NLP) is required to extract the meaningful terms from the unlabeled contents, to recognize their type and context, and then finally to place the processed terms in the target PICO format. The abstract flow of PICO construction from EHR data is depicted in Figure 4.

The following natural language preprocessing steps are applied in a pipeline: (1) the text is broken into tokens with a space delimiter; (2) stopwords of English language are removed; (3) case of the letters is changed to lower; and (4) the words are stemmed to their root words using porter stemming.

The stemmed words are mapped to the PICO format using salient term identification (STI) algorithm explained in the following section. After finding out a concept in the standard vocabulary of Systematized Nomenclature of Medicine-Clinical Terms (SNOMED-CT) implemented through the Unified Medical Language System (UMLS) vocabulary service application programming interface (API), the algorithm further finds out the semantic and entity types of the identified concepts. Finally, the identified concepts are mapped to PICO-corresponding slots. For example, because female belongs to population group, it is mapped to the P slot of PICO.

#### Scenario 2: An Automatic Query Construction From the Clinical Decision Support System Knowledge Base

In this scenario, the contents of the query are extracted from the rules of a CDS KB. The rules are actuated against a particular decision. The extraction process relies on the representation scheme of knowledge. Of these schemes, we elaborated on the following 2 schemes for the mapping of rules to PICO: plain production rules (if-then) and the HL7 medical logic modules (MLM) [21]. We designed a general model that could be used for any of the representation schemes. According to the proposed mapping model, different parts of a rule are mapped to PICO as described in equation 1:

PICO=D ∩ A ∩ *P* (1)

**Figure 5:**
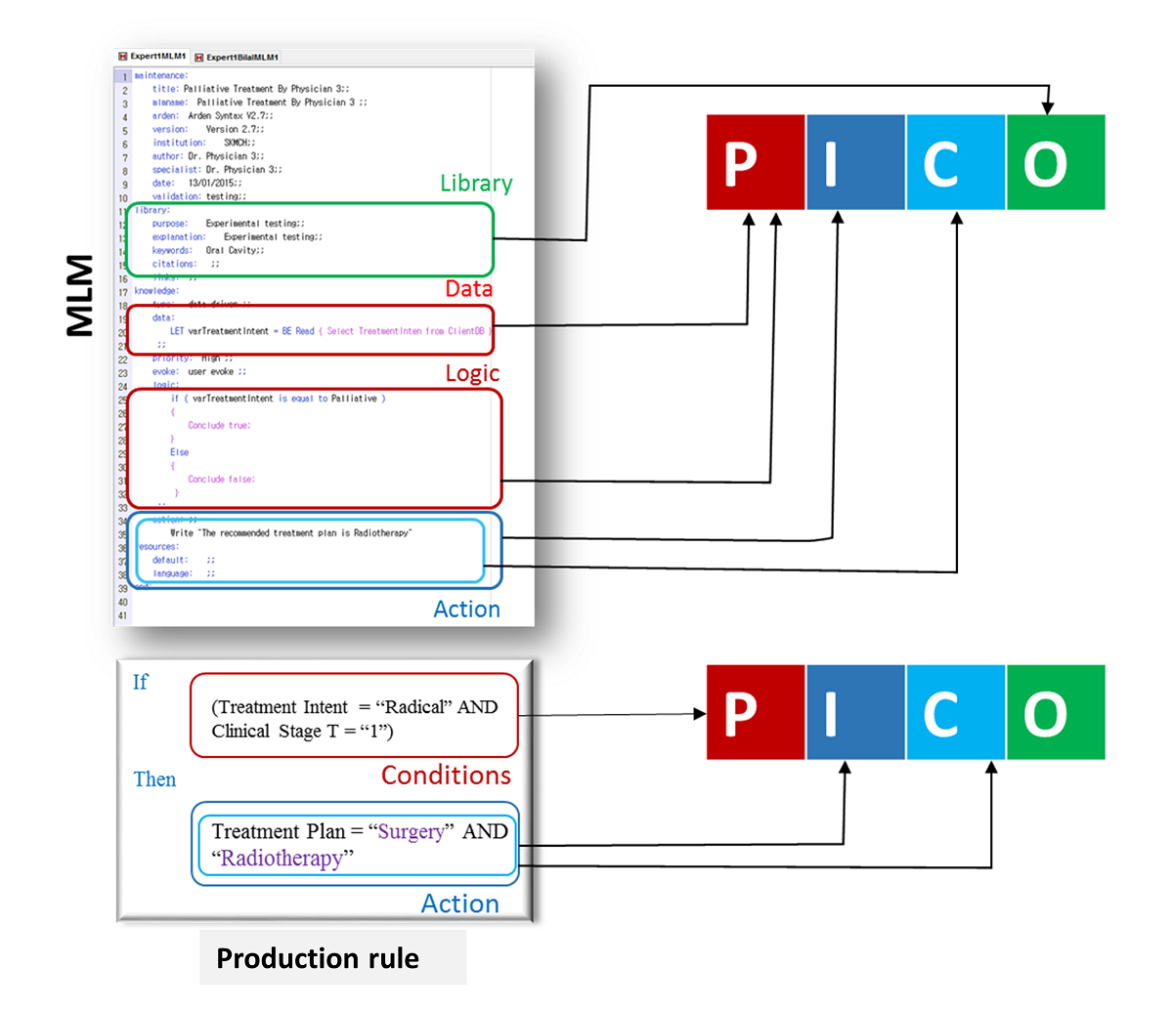
An example of Patient/problem, Intervention, Comparison, and Outcome mapping from the HL7 medical logic module and the production rule. MLM: medical logic module; PICO: Patient/problem, Intervention, Comparison, and Outcome.

In equation 1, D represents the set of elements in the data part of a rule, A is the set of elements in the action part, and P shows the purpose of a rule. More specifically, D maps to P of PICO, A maps to both I and C, and P maps to O of PICO. For clarity, Figure 5 is provided to describe the mapping from rules to PICO using MLM and the plain production rules as an example. In the scenarios where there is lack of information to get outcome information from the input, some elements of PICO can be unmapped. For example, in the scenario of production rules, the O part of PICO remains unmapped.

#### Scenario 3: Constructing a Query From the Multimodal Sensory Data

**Figure 6:**
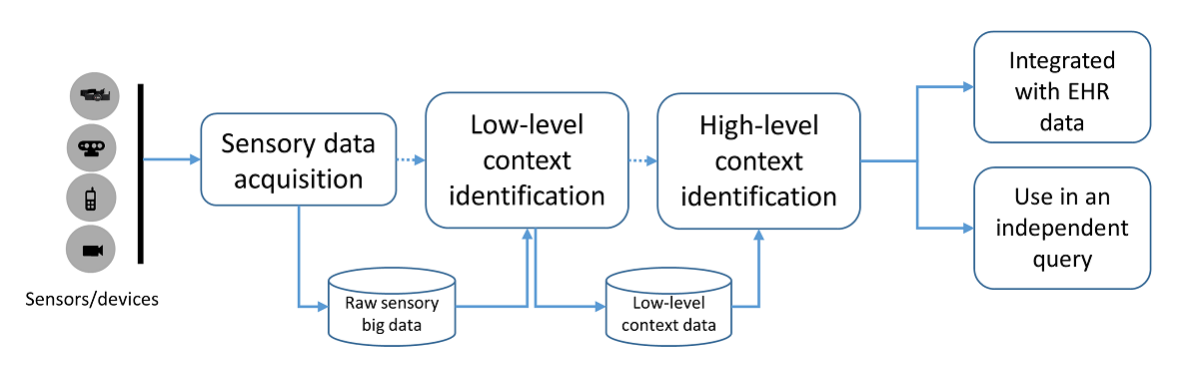
A dataflow diagram of the raw sensory data acquisition and the context identification. EHR: electronic health record.

This scenario is more applicable to participatory health management, where user activity, diet, sleep, and other related information are acquired through different sensors and devices. The information from these sensors and devices are collected independently through their independent clocks with an associated time stamp. A logical clock is required to identify the data origination at the same time [22]. After synchronization, the raw data need to be labeled and persisted for other services to consume. Using the labeled data may require further processing to determine the high-level context for the appropriate usage in the query. For instance, if a user is doing a set of activities, such as walking, running, or lying down, in an adjacent frame of time, it may refer to a high-level context of exercise. In one of our preliminary work on the project of Mining Minds [23], we have developed different models for context recognition both at the lower and the higher levels on the basis of data curated from different sensors. The dataflow of the raw sensory data is briefly illustrated in Figure 6. It must be noted that the contextual information determined from the sensory data could either be used in an independent query or used as a subset information of a query constructed from the EHR data as noted in previous subsections. There could be scenarios to combine methods of the abovementioned 3 scenarios and construct a single query depending on the availability of the data and the user needs.

### Concept Matching for Term Inclusion and Exclusion

**Figure 7:**
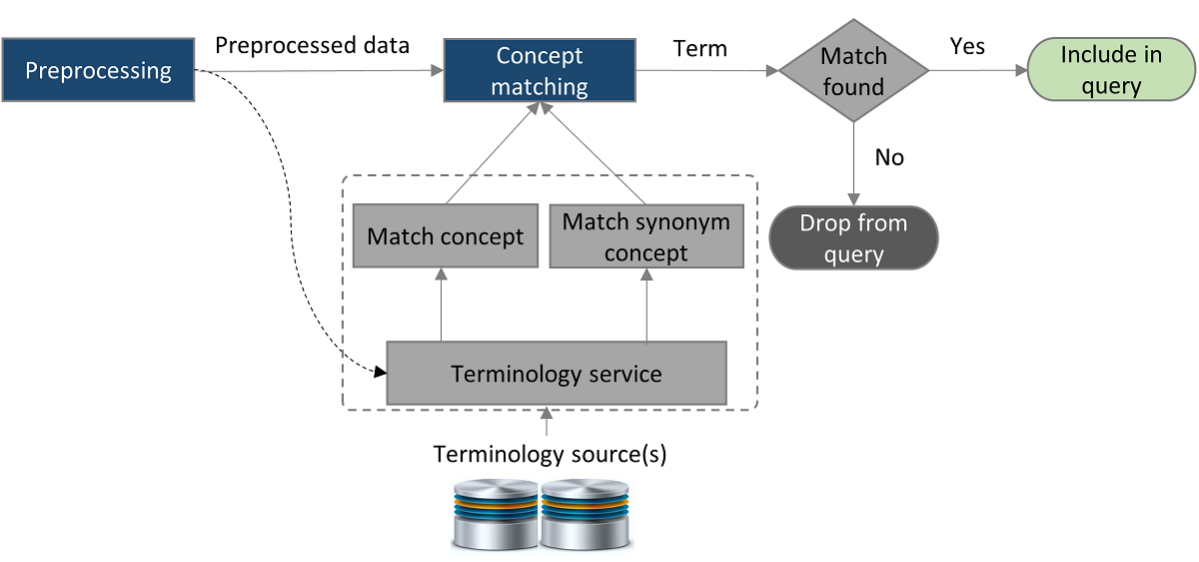
The salient term identification algorithm.

At the time of term extraction from the EHR data, it is important to include only pertinent and important terms. We developed the STI algorithm to filter out the less effective terms from the user question. The STI is a weight-based algorithm that finds an input term in a terminology source (SNOMED-CT/UMLS) and provides weight according to the matching level, such as exact match, partial match, and synonym match. The steps of the STI algorithm are described in Figure 7. According to this algorithm, if a term finds an exact match, it gets more weight (w=1.0) compared with the partial match and synonym match (w=0.5). The algorithm is formally represented in algorithm 1 in Multimedia Appendix 1.

### Standardization and Context Enrichment

**Figure 8:**
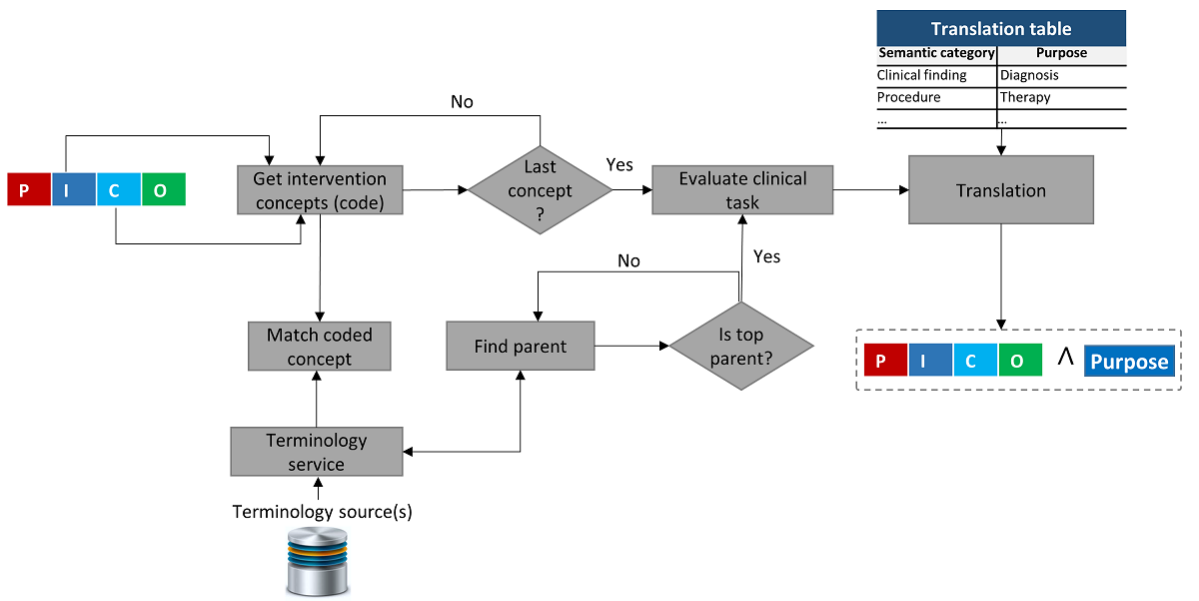
The steps of query type identification algorithm. PICO: Patient/problem, Intervention, Comparison, and Outcome.

It is not mandatory to use standard form of the terms; however, it is important to infer the overall intentions of the user from the query. In other words, the standardization helps to determine the users’ interest in obtaining results that are related to the diagnosis, the treatment, or the prognosis. In addition, it helps in understanding the meaning of a particular term precisely and helps avoid confusion. To achieve standardization of the terms and determine the purpose of the query, a terminology source, preferably the UMLS, could be used. We must remember that the function of standardization is achieved at the time of executing the STI algorithm. The purpose of the query, also known as query type, is determined from identifying the semantic types and entity types of the concepts used in the I and/or C parts of the PICO. As described in Figure 8, the concepts extracted from the PICO I and/or the C parts are used to determine their parent concepts with the help of the terminology services. After getting translated from the translation table for the parental term, the inferred translated concept (diagnosis/treatment/prognosis) is attached to the PICO query. During implementation of the query to run on the PubMed, the purpose term or the query type is used as a clinical filter for an increased recall [15].

### Query Optimization and Searching

**Figure 9:**
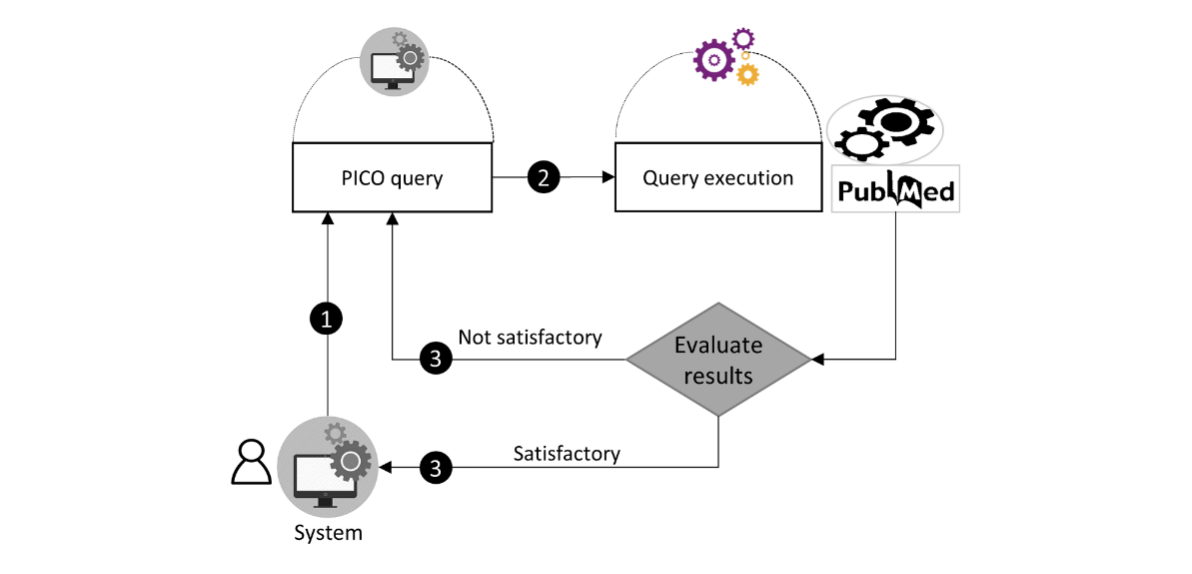
The query optimization process flow. PICO: Patient/problem, Intervention, Comparison, and Outcome.

A well-built structured query may be unnecessarily too lengthy to return absolutely no results or too short to return too many results. In this case, a query optimization technique is necessary to balance the resultant set. If there is a query that occasionally fails to retrieve any results, the query is optimized to exclude the least important term from a list of terms. As a general guideline, the P and I set of terms in PICO is the core, and they are considered comparatively more important than C and O. On the basis of this theory, we designed an optimization strategy, which is illustrated in Figure 9, to exclude a term from the least important parts one by one unless we retrieve an acceptable set of publications. For instance, if there are 4 terms ANDed in a query belonging to one of each PICO element and by running that query returns 0 records, we remove the C term first and check if the resultant set satisfies a threshold; if that is the case, we execute the query and continue the process further; otherwise, we remove the O term in the next cycle.

Even if the query terms consist of only P and I of PICO but are too many to retrieve any results, we can use the weights determined by the STI algorithm and remove the least effective term(s). The final optimized query was executed on biomedical literature and used the retrieved articles for further processing. For this research, we used the PubMed service to access the biomedical literature.

### Quality Evaluation

Articles retrieved using the PubMed built-in search strategy could possibly include quality, nonquality, or less quality articles that need to be segregated before presentation to the user. For this very reason, we designed quality parameters that need to be checked so that only quality contents come forward to be read by the users.

### Guidelines for Using the Gold Standard

We learned from previous studies that the Clinical Hedges database [24], which was developed by the Hedges Group at McMaster University, can be used as a gold standard dataset. Clinical Hedges was initially employed to develop and evaluate the CQ filters [15]. It is also used for ML approaches that identify the scientifically sound PubMed clinical studies [17]. The database consists of 50,594 MEDLINE articles published in 170 clinical journals, of which 49,028 articles are unique. All the articles are manually annotated by a team of specialized experts, and they classified the articles across the following 4 dimensions: format (O=original study, R=review, GM=general and miscellaneous articles, and CR=case report), human health care interest (yes/no), scientific rigor (yes/no), and purpose (diagnosis, etiology, prognosis, treatment, economic studies, reviews, and clinical predication guides). The primary purpose of creating the database was to evaluate whether each study was scientifically sound or not using the criteria for the treatment interventions, which include clinically relevant outcomes, random allocation of study participants, and at least 80% of the follow-up of study participants.

### Selection of the Search Strategy

One of the issues for common users and researchers is to choose an appropriate search strategy to satisfy their information needs. None of the state-of-the art search strategies could be considered ideal in all situations. We provide a common standpoint and recommendations to opt for a strategy based on the users’ needs and rationale. We divide the set of approaches in 3 groups: (1) PubMed search strategies that include mainly CQ, (2) the ML approach, and (3) the DL approach. We also categorize the user information needs based on recall, precision, and recentness, which is the instant availability of a study. Table 2 provides the performance evidence of the existing state-of-the-art approaches, whereas Table 3 provides the recommendation of using an approach on the basis of given rationale.

Regarding the third criteria, such as the recentness, we elaborated the abovementioned approaches on a delay factor. As both the PubMed CQ and the mentioned ML approaches use a Medical Subject Headings (MeSH) filter in the search strategies, they encounter problems classifying the most recent articles. The mean delay in the MeSH indexing per journal was recorded as 162 days. The DL neural network considers only the title and the abstract features and has no dependency on the MeSH terms and does not encounter any delays to evaluate even the most recent studies.

**Table 2:** Average recall and precision of different search strategies.

Approach	PubMed Clinical Queries (broad)	Machine learning	Deep learning	Reference
Average recall	98.4	91.4	96.9	Reported in the study by Perez-Rey et al [10] and Del Fiol et al [19]
Average precision	22.4	86.5	34.6	Reported in the study by Perez-Rey et al [10] and Del Fiol et al [19]

**Table 3:** Recommendation of the search strategy in a given rationale.

Rationale	Recommendation
User top priority is high coverage (recall)	PubMed Clinical Queries
User top priority is to get precise results (precision)	Machine learning classification approach
User top priority is more recent studies	Deep learning neural network approach

### Feature Engineering and Corpus Preparation

**Figure 10:**
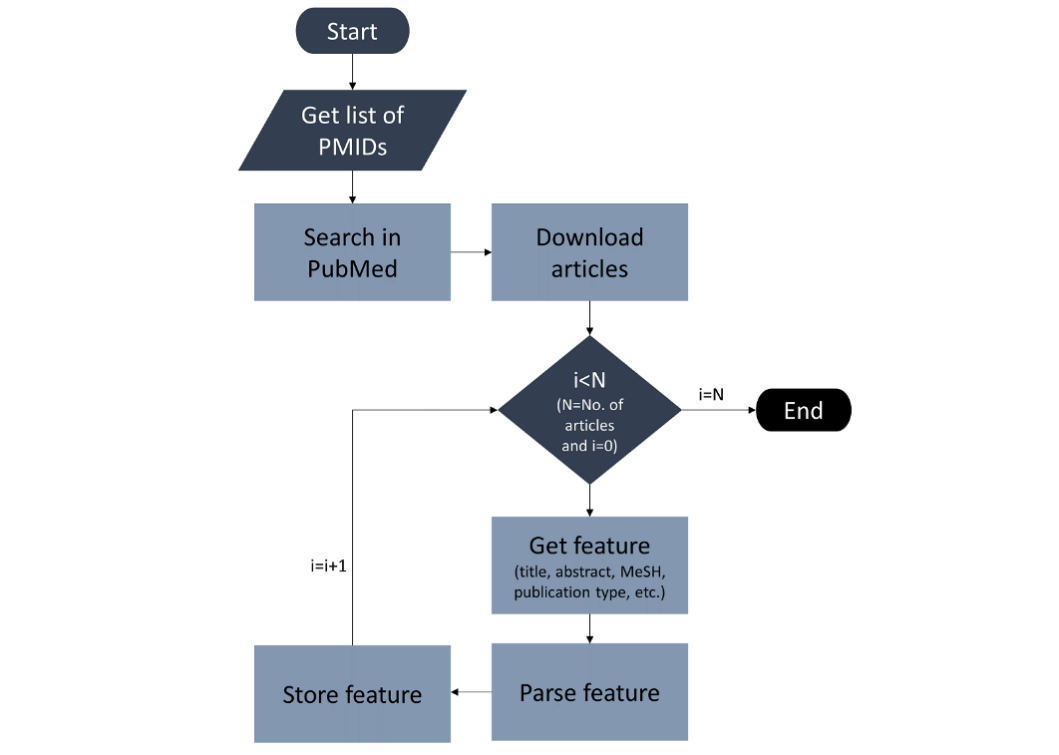
The article downloading and parsing algorithm. MeSH: Medical Subject Headings; PMIDs: PubMed Identifiers.

Irrespective of the strategy, we need to engineer the features and prepare a corpus to run ML or DL classifiers. We used the Clinical Hedges dataset and acquired the PubMed identifiers, which we posted to create a custom database on PubMed through the Entrez Post service method of the Entrez Programming Utilities API, and we searched the publications using the eSearch service for the Entrez Fetch (eFetch) service by enabling the history and the environment variables to yes. The eFetch function was used to download the searched articles. The downloaded records were programmatically parsed to obtain the data for the data features, such as the title, the abstract, and the metadata features, which include the MeSH terms and the article type. The process of downloading and parsing the articles is described in Figure 10.

We engineered 2 sets of features, which included the data features and the metadata features. The data feature vector was created by tokenizing the titles and abstracts, changing the case to lower, eliminating the stop words, stemming the words using the porter stemmer, and filtering the tokens by lengths. Unlike the data features, the metadata features were created by applying only tokenization and case transformation because there was no need to remove stop words and stemming.

### Extended Quality Recognition Model

**Figure 11:**
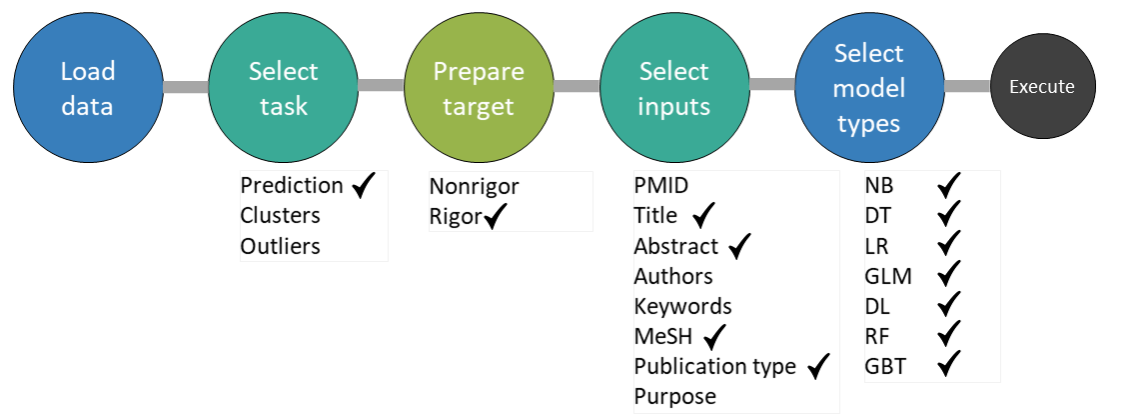
Automodel steps and parameter settings. DL: deep learning; DT: decision tree; GBT: gradient boosted trees; GLM: generalized linear model; LR: logistic regression; MeSH: Medical Subject Headings; NB: Naïve Bayes; PMIDs: PubMed Identifiers; RF: random forest.

Previously, we worked to identify the relevant and quality articles using the ML approach and developed a model called quality recognition model (QRM) [25]. The QRM was a binary classification model with the following 2 classes: rigor and nonrigor, where rigor and nonrigor represent the quality and nonquality articles, respectively. It was tested with 4 different ML algorithms, including Naïve Bayes (NB), k-nearest neighbor (kNN), decision tree (DT), and support vector machine (SVM). For this study, we extended the QRM with inclusion of DL model and ensemble technique to get a better performance. With the advent of automodel feature in the data science tool RapidMiner Studio 9.0.003 [26], it is rather more expedient to opt for an efficient model in a range of applicable models. Using the same dataset that we previously used for building QRM, the RapidMiner automodel function proposed 7 algorithms, including NB, DT, logistic regression, generalized linear model (GLM), random forest, gradient boosted trees, and DL. We keep consistency in the feature set selection, similar to the previous model of QRM. The automodel function allows us to intervene in the settings of parameters at different steps including the load of dataset, selection of the task, preparing a target, selection of the input features, and model types to execute for getting the final results. We described these steps in Figure 11 by highlighting our selection among alternatives.

We use the automodel function to select the individual learners to build our ensemble model to acquire high accuracy as compared with the performance of individual learners. We first experimented with the individual models proposed by automodel function. Later, we develop an ensemble learner over the individual learners. In the first layer of assembling, we use AdaBoost learner, whereas on the second layer, we use ensemble voting (stacking model) with a sampling type of automatic, which uses stratified sampling per default. However, if the example set does not contain a nominal label, shuffled sampling is used instead. The split was relative with a split ratio of 0.7.

### Ranking and Summarization

A potential problem that clinical user face is the management of the results set to identify, appraise, and synthesize the best available evidence to answer the clinical question in the best possible manner. In all this process, a lot of manual effort is required to extract the data to make a summary, and it is also subjected to error [27]. Moreover, the context of the user may change the default ranking of an article to bring it to the top or take it to the bottom. Some of the existing approaches use a grading mechanism of ranking the articles based on the strength of the contents [18,28]. The need to develop a ranking mechanism that is more patient-centered rather than only evidence-centered is needed. In addition, the model needs to consider the user’s context in addition to the articles’ strength and quality.

To address these issues, we conferred our previous work [25] and made possible extensions to provide guidelines for using an appropriate model in a given clinical situation. We devised a cross-context evaluation strategy that involves crossly matching 2 contexts, such as user contexts and evidence contexts. User contexts have multiple elements, such as basic information, which shows the user educational level. The background is the experience of the user, and the goal shows the short-term learning or the long-term learning. The interest represents the preferences, and the learning style is the pattern of user learning, such as textual and visual. On the other hand, an evidence context includes the article meta-features or properties, such as the publication type, the publication avenue (eg, journal and book), and the year of publication.

We devise a cross-context evaluation method that accumulates contextual parameters of both user and evidence contexts. User context parameters are represented as C1, C2, ..., Cn, whereas the evidence contextual parameters, which are properties of a publication, are represented as P1, P2, ..., Pn. These 2 sets of contexts are aggregated first vertically and then horizontally to reach to the final grading of a publication as H=high, M=medium, L=low, and U=unknown. In other words, the algorithm first finds the aggregate value of each column, such as the highest value of all the cells using the majority vote procedure, and the process is repeated for all the cells. The example described in Table 4 for a user with three contexts and a publication with two properties, the final context value is calculated for a given publication-x by learning the highest value from the aggregate contexts AggCtx-1 and AggCtx-2. The value H, M, L, and U are learned from the ranking values assigned to each of the article types described in Table 5.

**Table 4:** The context aggregation for article ranking.

User context	Publication-x context	
	P1	P2
C1	H^a^	M^b^
C2	L^c^	M
C3	H	H
Aggregate context	AggCtx-1=H	AggCtx-1=M

^a^H: high.

^b^M: medium.

^c^L: low.

**Table 5:** Values of publication types ranking and grading.

Publication types	Ranking	Grade value
Systematic reviews	1	H^a^
Meta-analysis of RCTs^b^	1	H
RCTs	3	H
Meta-analysis of CTs^c^	4	M^d^
Systematic review of CTs	5	M
CT	6	M
Cohort study/case-control study/report	7	M
Guidelines	8	L^e^
Opinion	9	L
Observational study	10	L
Any other publication type	11	L

^a^H: high.

^b^RCTs: randomized controlled trials.

^c^CT: control trials.

^d^M: medium.

^e^L: low.

Finally, the evidence is formed from the set of the ranked articles, and it is presented in the form of a summary. As mentioned earlier, the manual summarization is a daunting task, and researchers have devised different methodologies to perform the automatic summarization of the articles. Bui et al [27] developed a computer-based ML and an NLP approach to automatically generate a summary of full-text publications by extracting the PICO values alongside the sample size and group size from the text. There are few other studies that proposed algorithms to detect PICO elements in the primary studies [29,30], which in term assisted the process of summarization.

**Figure 12:**
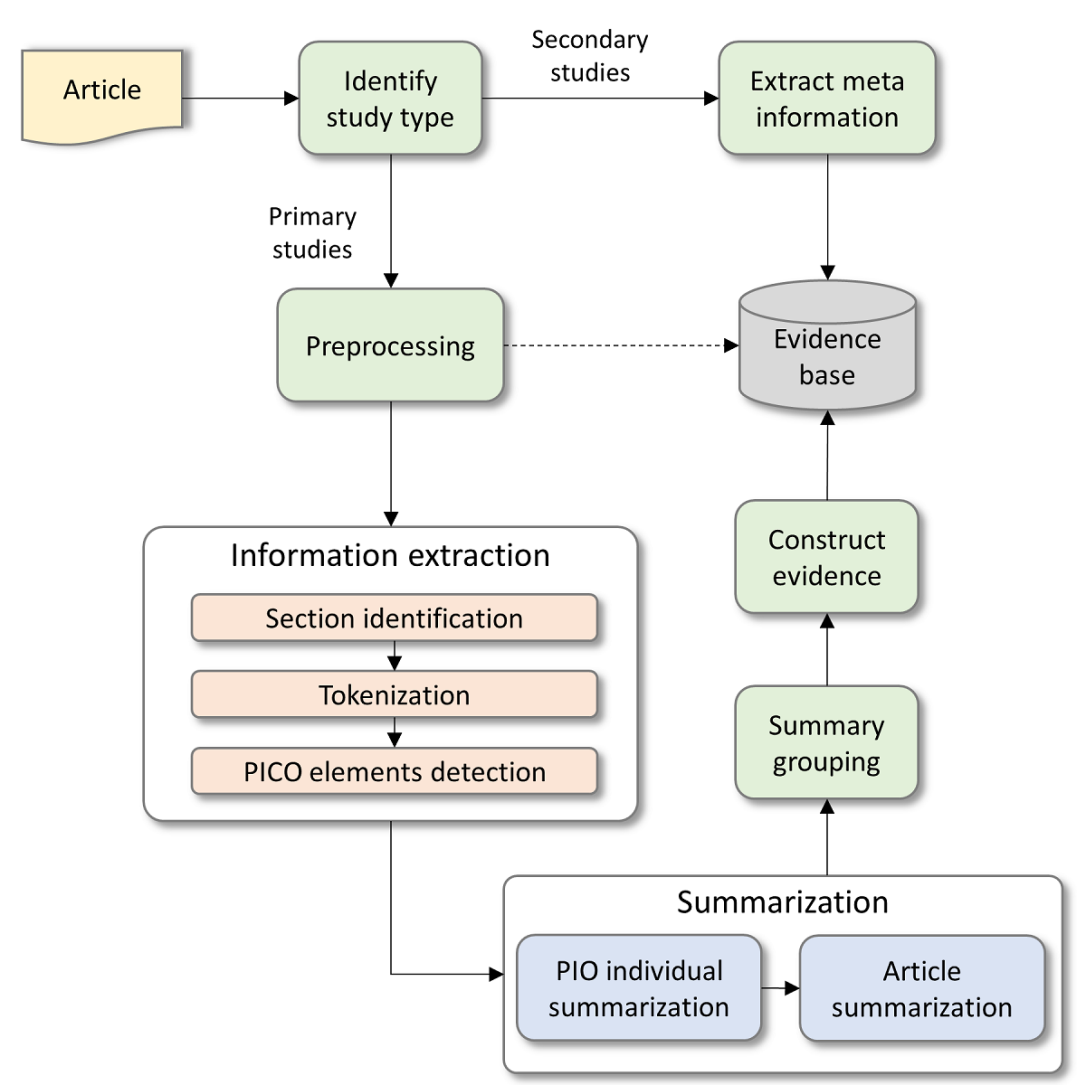
The framework for article summarization. PICO: Patient/problem, Intervention, Comparison, and Outcome; PIO: Patient/problem, Intervention, and Outcome.

We are convinced that the PICO-based approach assists the extractive method of automatically generating the summaries of articles. As a result, we followed the extraction of the PICO values from the text of an article. Before the extraction, we identify if the type of article is primary or secondary. The secondary articles such as systematic reviews (SRs) are formed from multiple primary studies, and they could be considered as evidence. However, the primary studies required summarization to form an evidence. We provide an abstract view of the proposed summarization system, as shown in Figure 12, to categorize articles as secondary and primary studies and construct summaries for the latter. The system works as each ranked article is taken as input and is distinguished as a primary study or secondary study. A secondary study is only processed to extract the meta information and is stored to the evidence base as an evidence. A primary study is preprocessed to convert the format from pdf to text in the first place. The text is passed to the information extraction module to identify sections and PICO elements in different section to generate individual summaries for P, I, and O elements of PICO. The element C is also an intervention, so a separate summary is not necessary; therefore, we consider it as a part of I during summarization. On the basis of individual summaries of Patient/problem, Intervention, and Outcome elements, a complete summary is constructed for the whole article. Individual articles’ summaries are grouped based on their similarities to form a single evidence, which is then stored to the evidence base by including the meta information associated with each article.

## Results

### Overview

As mentioned earlier, the focus of this study is to construct a PICO-compliant query from EHR data where there were 2 evident options to acquire query terms: structured data and unstructured data. One of the possible options that could be used for structured data is the knowledge rules of a CDSS, and any clinical scenario explained in simple English can be considered for the unstructured data case study. In knowledge rules, it is quite straightforward to identify the clinical concepts and map them to the PICO elements. However, unstructured data mapping to PICO requires quite a few steps to get the final PICO-based query, as discussed in the query construction section.

### Case Study—Query Construction From a Clinical Scenario

**Figure 13:**
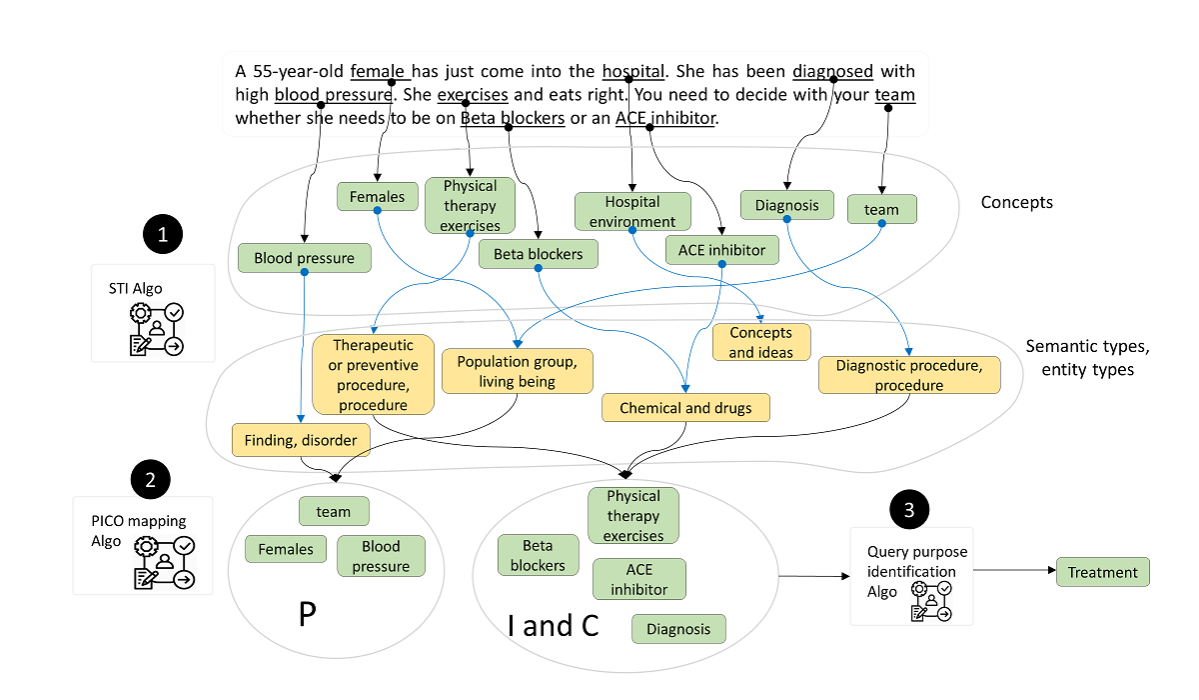
Results of a clinical scenario conversion to Patient/problem, Intervention, Comparison, and Outcome with query type (purpose). ACE: angiotensin-converting-enzyme; PICO: Patient/problem, Intervention, Comparison, and Outcome; STI: salient term identification.

Here, we present a clinical scenario and step-by-step outcomes of our proposed algorithms, mapping to PICO and finally a search query construction. The first step is to find important clinical concepts in the given clinical scenario followed by finding their semantic type and entity type. In the scenario shown in Figure 13, our proposed STI algorithm found out 8 concepts in the standard vocabulary of SNOMED-CT implemented through the UMLS vocabulary service API. For the identified concepts, we identified the semantic and entity types of the identified concepts. In the second step, the identified concepts are mapped to PICO-corresponding slots, such as female belongs to population group, so it is mapped to the P slot of PICO. Similarly, beta-blocker has the entity type of chemical and drugs, so it went to the I (intervention) slot of PICO. In the same way, we mapped the rest of the concepts. Finally, in the third step, we find the type of the query among diagnosis, treatment, prognosis, or etiology. Looking at the semantic/entity types of majority of the concepts in I and C, the algorithm concluded with the query type as treatment. Using the logic of searching query construction as described earlier, we will get the query as follows:

q=female blood pressure and (beta-blocker or ACE inhibitor) (2)

This query *q* is passed to our searching algorithm to search for the publications using the PubMed CQs utility.

### Quality Evaluation

The existing QRM was tested using multiple ML approaches, which included the SVM, the DT, the kNN, and the NB. The results were reported in our previous study [25], where the SVM algorithm has performed better than other algorithms. The automodel employed algorithms produced varied results for different algorithms. As shown in Table 6, the gradient boosted trees (GBT) algorithm outperformed other algorithms with accuracy of about 84.98%, followed by GLM with accuracy of about 83.80% at the individual learning stage. To minimize the instances of wrong classification, we tested ensemble method using AdaBoost on top 3 individual learners. We noticed that GBT was still on the top, with an increase of about 4% accuracy jumping from 84.98 to 88.50. The performance of GLM was slightly increased by about 1%, whereas the DL performance with AdaBoost was increased by about 9% accuracy from 75.48 to 84.57. In the final model, we use a second level of ensemble over AdaBoost (GBT) and GLM and obtained about 3% improved accuracy of 90.97%. Moreover, area under the curve (AUC) value of the E-QRM was noted as 0.989, whereas for AdaBoost (GBT), it was 0.950, followed by AdaBoost (DL) with AUC value of 0.921.

It is important to note that the experiments are performed using RapidMiner Studio version 9.3.001, which is an improved version of the descendants. All the models, particularly the automodel, may generate a different set of results even on the same data because of the changes in operators for the possible improvement from version to version.

**Table 6:** Extended quality recognition model performance overview using different algorithms.

Algorithm/criteria	F measure	Precision	Accuracy	Area under the curve
Naïve Bayes	0.53	0.42	0.64	0.53
GLM^a^	0.72	0.82	0.84	0.89
Logistic regression	0.46	0.39	0.62	0.63
Decision tree	0.09	0.50	0.70	0.50
DL^b^	0.57	0.59	0.75	0.78
GBT^c^	0.75	0.77	0.85	0.87
AdaBoost (GLM)	0.73	0.78	0.85	0.82
AdaBoost (DL)	0.72	0.79	0.85	0.92
AdaBoost (GBT)	0.79	0.85	0.89	0.95
Extended quality recognition model	0.85	0.83	0.91	0.98

^a^GLM: generalized linear model.

^b^DL: deep learning.

^c^GBT: gradient boosted trees.

### Ranking and Summarization

**Figure 14:**
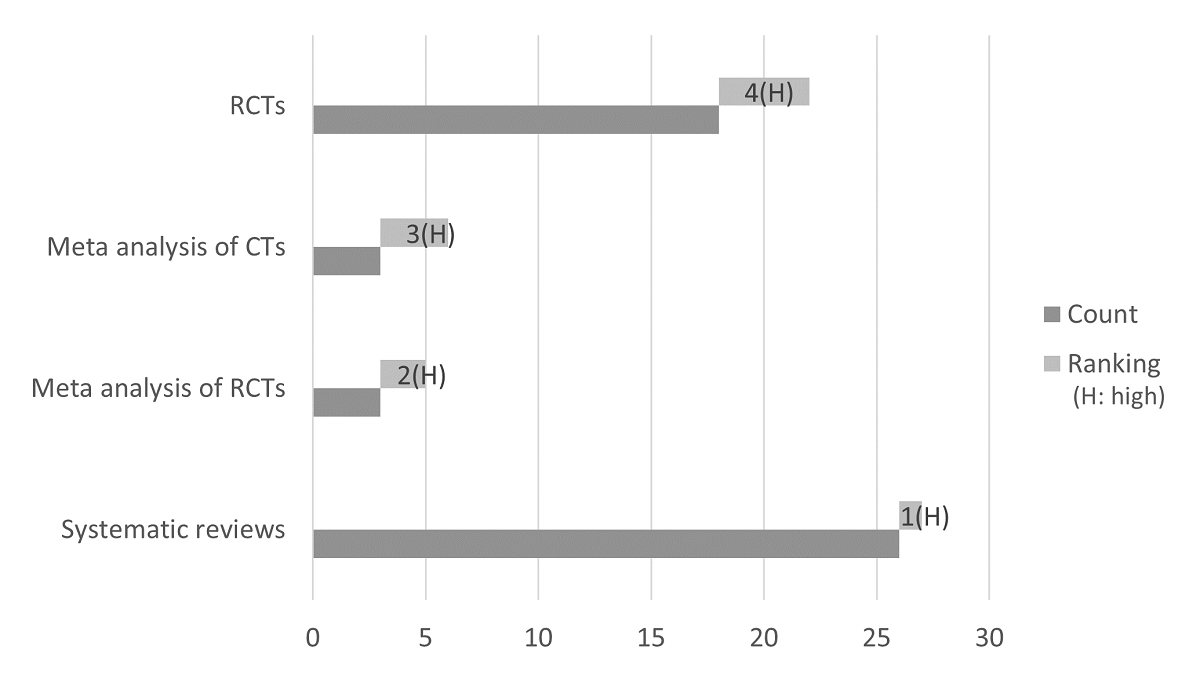
Top 50 publication types with rank and grade values. CT: control trials; RCTs: randomized controlled trials.

Linking to our previous study results for contextual ranking, we determine the ranks for the studies retrieved against the query in equation 1. The query returned overall 5243 articles, of which 5217 were primary studies and other 26 were SRs. In the primary studies, the highest count of 5187 was assigned to randomized controlled trials, and the rest were distributed among meta-analysis (6), observational studies (5), case reports (2), and others (17). On the basis of the ranking assigned to different publication types in Table 5, the rank and grade values are produced for the selected set of top 50 out of 5243 publications, as shown in Figure 14.

## Discussion

### Principal Findings

The main findings of this study include the design of a comprehensive framework encompasses methods of automatic query construction using PICO, the quality assessment using data-driven approaches, and the ranking of studies using contextual aggregation matrix. Compared with the existing QRM, our high-impact ensemble classifier E-QRM obtained significantly improved accuracy (1723/1894, 90.97% vs 1462/1894, 77.21%). Moreover, the proposed work has the significance and the implication in multiple domains, and here, we present a few of those applications.

### Significance in Evidence-Based Medicine

Research evidence is one of the components of EBM. The proposed literature curation framework is best fit to locate and incorporate the best evidence from the biomedical literature in PubMed to support the evidence-based medical decisions. This work provides a comprehensive set of methods to bring automation to different levels of searching scientific publications, ranging from query construction, quality recognition, and summarization and ranking. The implementers of the EBM can extend and integrate the proposed framework with the health system to use in their daily clinical practice.

### Significance in Precision Medicine

Precision medicine is a multidisciplinary approach, which involves genetic characteristics along with clinical and environmental behavior for making a precise decision. There is an opportunity to study how observational studies and clinical trials can be used in conjunction to improve health outcomes in real-world practice settings [31]. This work can greatly contribute to find relevant phenotypes and genetic information precisely from Web-based biomedical resources, including the GenBank [32], MedGen [33], ClinVar [34], and other databases. A set of studies have investigated and developed tools for the evaluation of phenotype candidates using online medical literature [35,36]. The work discussed in this study provides flexibility to apply it to find phenotypes and genome-related clinical trials and evaluate their strength.

### Significance in Clinical Decision Support Systems

The CDSS decisions are more trustable if relevant evidence from external sources is timely integrated. The proposed framework could be integrated with the existing CDSS by connecting the query part with the output of the CDSS. The concept of health level seven clinical decision support hooks (HL7 CDS Hooks) [37,38] was very recently extended to include evidence information retrieved from scientific literature. Moreover, the existing CDSSs could be extended to adapt the scientific research evidence in real time.

### Significance in Medical Education

Students and researchers require to educate themselves on the existing work from experts. An efficient way to access the existing research work is to implement a system that assists them in a meaningful manner. The proposed framework is capable of providing a unique opportunity to obtain the best evidence in less time and with a higher level of accuracy. Researchers need guidance on whether they have to apply a new method of intervention and at what cost. Patients do need literature to study and compare their conditions with other similar patients and find out about the outcomes of the interventions on other patients.

### Limitations of the Work

The summarization research work is yet to mature; therefore, its results are not reported in this study. We have a plan to continue our investigation further to design automated methods and guidelines for the construction of summarization such as designing methods to generate a summary of different articles for the formation of a consolidated evidence. Moreover, we are also interested in investigating the strategies for discovering knowledge from the evaluated quality articles.

### Conclusions

There is a great demand for consultation of external clinical evidences to be considered in a complex clinical decision-making process, particularly when internal evidences are insufficient. In addition, medical researchers, students, and patients use them for education, training, and self-awareness about their health problems, respectively. To satisfy these users’ needs and desires, we proposed a comprehensive framework for automated curating of biomedical literature, which facilitates the task of bringing a quality research evidence intelligently to the users’ desk to assist the users in answering clinical questions and fulfilling their informational needs. We presented a set of methods and guidelines to automate the process of curating biomedical literature at 3 levels: query construction, quality recognition, and ranking and summarization supported with sample results. This proposed automated framework is expected to improve the overall efficiency of clinical staff and researchers in terms of time and effort. However, the proposed system needs to be thoroughly tested on multiple domains before adoption. Our future work will focus on the optimization of quality evaluation and ranking and summarization. The final approval of the evidence by a human is crucial to avoid interpretations if wrong decisions are made by the system.

## Appendix

Multimedia Appendix 1 Algorithm: salient term identification.

## Abbreviations

API: application programming interface
AUC: area under the curve
CDSS: clinical decision support system
CQ: Clinical Queries
DL: deep learning
DT: decision tree
EBM: evidence-based medicine
EBP: evidence-based practice
eFetch: Entrez Fetch
EHR: electronic health record
E-QRM: extended quality recognition model
GBT: gradient boosted trees
GLM: generalized linear model
HIS: health care information system
IITP: Institute of Information & Communications Technology Planning & Evaluation
KB: knowledge base
kNN: k-nearest neighbor
MeSH: Medical Subject Headings
ML: machine learning
MLM: medical logic modules
MSIT: Ministry of Science and ICT
NB: Naïve Bayes
NLP: natural language processing
PICO: Patient/problem, Intervention, Comparison, and Outcome
QRM: quality recognition model
SNOMED-CT: Systematized Nomenclature of Medicine-Clinical Terms
SR: systematic review
STI: salient term identification
SVM: support vector machine
UMLS: Unified Medical Language System
