# Supplementary material for: Impact of Automatic Query Generation and Quality Recognition Using Deep Learning to Curate Evidence From Biomedical Literature: Empirical Study
Source: JMIR Med Inform. 2019 Dec 9;7(4):e13430. doi: 10.2196/13430 (PMC6928703; doi:10.2196/13430)
Supplement: Multimedia Appendix 1 [file medinform_v7i4e13430_app1.docx]

# Multimedia Appendix 1: STI Algorithm

| **Algorithm 1.** Salient term identification (STI) |
| --- |
| **Begin**  **inputs:** $\boldsymbol{C}-\left\{ p, i,c,o \right\};$ *# set of query terms and each term with its initial weight = 0.0 and limit*  **output:**$\boldsymbol{R-}$ $\left\{ r_{1}, r_{2},\ldots, r_{n} \right\};$ *# the set of qualified PICO terms*   1. ***Initialize* query** 2. $\boldsymbol{foreach}pico in \boldsymbol{C}$ 3. terms = *pico.getTerms();* 4. *type = pico.getType () # returns type of concept e.g. P, I, C or O* 5. $\boldsymbol{foreach}term in \boldsymbol{terms}$ 6. ***if*** **exact_matched(**term**);** then *# if concept is exact matched in standard vocabulary* 7. *term.weight = 1.0* 8. ***elseif* partial_matched(**term**)** 9. *term*.*weight = 0.5* 10. ***endif*** 11. ***endfor*** 12. ***sort_by_weight_desc(****pico****)*** 13. ***query.concate( build_optimized_query(****pico, type****) )*** 14. ***endfor*** 15. *R = execute_optimized_query(query)*   **End**  **Procedure build_optimized _query(T, Type)**  **Begin**  **inputs:** $\boldsymbol{T}- \left\{ c_{1}, c_{2},\ldots, c_{n} \right\}$ *# set of terms of sorted by weight in DESC order*  **Type** *# the type of concept e.g. P, C, I or O*  **output:**$\boldsymbol{Q-}optimized pubmed query$   1. *counter* = 0; 2. ***if*** count_exact_match(**T**) greater*_than* 0 then # e.g. either exactly or partial matched 3. $\boldsymbol{foreach c}in \boldsymbol{T}$ 4. ***if* c.**weight ***equals*** 1.0 ***AND*** *counter* ***lessThan*** c.*limit* 5. **Q**.concate(“(“).concate(***c***.value) 6. **Q**.concate(“OR”) 7. **Q**.concate(***c***.matched_value).concate(“)”) 8. ***Q.****concate(“AND”)* 9. ***increment*** *counter* 10. ***endif*** 11. ***endfor*** 12. ***else*** 13. ***If*** *Type* ***notEquals*** *C* ***OR*** *O* 14. **Q**.concate(p.value) 15. ***endif*** 16. ***endif*** 17. ***return*** Q *# optimized query*   **End** |
